# Phenotypic and molecular characterization of pyrazinamide resistance among multidrug-resistant *Mycobacterium tuberculosis* isolates in Ningbo, China

**DOI:** 10.1186/s12879-021-06306-1

**Published:** 2021-06-25

**Authors:** Yang Che, Dingyi Bo, Xiang Lin, Tong Chen, Tianfeng He, Yi Lin

**Affiliations:** 1 Institute of Tuberculosis Prevention and Control, Ningbo Municipal Center for Disease Control and Prevention, Ningbo, 315010 Zhejiang China; 2 Institute of Tuberculosis Prevention and Control, Haishu Municipal Center for Disease Control and Prevention, Ningbo, Zhejiang China; 3 Center for Health Economics, Faculty of Humanities and Social Sciences, University of Nottingham, Ningbo, Zhejiang China

**Keywords:** Multidrug-resistant, *Mycobacterium tuberculosis*, *pnc*A gene, Resistance

## Abstract

**Background:**

Detection of pyrazinamide (PZA) resistance in *Mycobacterium tuberculosis* (TB) patients is critical, especially in dealing with multidrug-resistant *Mycobacterium tuberculosis* (MDR-TB) case. Up to date, PZA drug susceptibility testing (DST) has not been regularly performed in China. The prevalence and molecular characteristics of PZA resistance in *M.tuberculosis* isolates, especially MDR-TB have not been studied in Ningbo, China. This study aimed to analyze the phenotypic and molecular characterization of PZA resistance among MDR-TB isolates in Ningbo.

**Methods:**

A total of 110 MDR-TB isolates were collected from the TB patients who were recorded at local TB dispensaries in Ningbo. All clinical isolates were examined by drug susceptibility testing and genotyping. DNA sequencing was used to detect mutations in the pncA gene associated with PZA resistance.

**Results:**

The prevalence of PZA resistance among MDR-TB strains in Ningbo was 59.1%. With regard to the history and the outcome of treatments among MDR-TB cases, the percentages of re-treated MDR-TB patients in the PZA-resistant group and of successful patients in PZA-susceptible group were significantly higher than the ones in the PZA-susceptible group and in the PZA-resistant group, respectively (*P* = 0.027, *P* = 0.020). The results showed that the resistance of streptomycin (67.7% vs 46.7%, *P* = 0.027), ethambutol (56.9% vs 33.3%, *P* = 0.015), ofloxacin (43.1% vs 11.1%, *P* = 0.000), levofloxacin (43.1% vs 11.1%, *P* = 0.000), pre-XDR (pre-Xtensively Drug Resistance) (38.5% vs 15.6%, *P* = 0.009), were more frequently adverted among PZA-resistant isolates compared with PZA-susceptible isolates. In addition, 110 MDR-TB was composed of 87 (PZA resistant, 78.5%) Beijing strains and 23 (PZA resistant, 21.5%) non-Beijing strains. Fifty-four out of 65 (83.1%) PZA-resistant MDR strains harbored a mutation located in the *pnc*A gene and the majority (90.7%) were point mutations. Compared with the phenotypic characterization, DNA sequencing of *pnc*A has sensitivity and specificity of 83.1 and 95.6%.

**Conclusion:**

The mutations within *pnc*A gene was the primary mechanism of PZA resistance among MDR-TB and DNA sequencing of *pnc*A gene could provide a rapid detection evidence in PZA drug resistance of MDR-TB in Ningbo.

## Background

Multidrug-resistant tuberculosis (MDR-TB) which is resistant to isoniazid and rifampin continues to be a great public health threat [1, 2]. World Health Organization (WHO) reported that about 0.48 million new cases of rifampicin-resistant TB, which 78% had multidrug-resistant TB, worldwide in 2018 were reported [3]. China has been the second burden in MDR-TB in the world with 66 thousands prevalent MDR-TB cases annually [3]. One recent report based on drug-resistant TB national survey in China showed that 7.1% of new TB cases and 21% of previously treated cases were MDR-TB [4]. The estimated incidence of TB was 0.04% residents in 2019 in Ningbo and the epidemic of MDR-TB has been considered one of a serious public health concerns in China because of its treatment failure [4, 5].

WHO recommends a treatment regimen including pyrazinamide (PZA) as cornerstone first-line anti-tuberculosis agent to the majority of MDR-TB patients [6, 7]. PZA as a pro-drug becomes toxic to *M.tuberculosis* under acidic conditions (PH = 5.5) requires conversion into its active form pyrazinoic acid with the enzyme pyrazinamidase, which was encoded by the *pnc*A gene [8, 9]. The main genetic mechanism of pyrazinamide resistance lies in the mutations within the 561-nucleotide *pnc*A gene [10, 11]. Because of its uniquely bactericidal effect, detection of PZA resistance in TB patients is critical, especially in dealing with MDR-TB case.

Up to date, PZA drug susceptibility testing (DST) has not been regularly investigated in China, thus, limited data has been updated reporting PZA resistance among MDR-TB isolates in the mainland of China [12, 13]. The prevalence and molecular characteristics of PZA resistance in *M.tuberculosis* isolates, especially MDR-TB have not been studied in Ningbo, China. The objective was to investigate the prevalence of PZA resistance among MDR-TB isolates in Ningbo and to analyze the characteristics of mutated *pnc*A gene conferring PZA resistance.

## Methods

### Study design

The first study of drug-resistant TB in the entire Ningbo from 2015 to 2017 by Ningbo Center for Disease Control and Prevention (CDC). A total of 1325 participants registering and diagnosed with TB at local TB dispensaries, were recruited in the present study and all the medical and related information were collected by well-trained nurses in the local hospitals. The isolates collected from TB patients were cultured on Lowenstein-Jensen (L-J) medium for 4–8 weeks and the culture with growing colonies were delivered to the Ningbo Tuberculosis Control Institute for further drug susceptibility testing. New cases were defined as patients previously receiving none or less than one month of anti-TB treatment. Re-treated cases were defined as patients previously receiving more than one month of anti-TB treatment. One hundred and ten consecutive MDR-TB patients out of 1325 patients with full medical and microbiological information were assessed for study eligibility.

### Drug susceptibility testing

Tests for susceptibility to four first-line anti-TB drugs and six second-line drugs were performed with the proportional method recommended by WHO [14]. The concentrations of drugs in L-J medium were as follows: isoniazid (INH), 0.2 μg /mL; rifampicin (RIF), 40 μg /mL; ethambutol (EMB), 2 μg /mL; streptomycin (SM), 4 μg /mL; ofloxacin (OFLX), 2 μg /mL; levofloxacin (LVX), 2 μg /mL; kanamycin (KAN), 30 μg /mL; amikacin (AMK), 30 μg /mL; capromycin (CAP), 40 μg /mL; protionamide (PTO), 40 μg /mL and p-aminosalicylic acid (PAS), 1 μg /mL [14]. The PZA drug susceptibility testing was performed with a Bactec MGIT 960 system and critical concentration was 100 μg /mL [15]. Quality control was performed during DST using the H_37_RV reference strains.

### Definitions

#### Drug-resistant *Mycobacterium tuberculosis* types

MDR-TB was defined as those resistant to both isoniazid and rifampicin. Pre-XDR TB was defined as MDR-TB additionally resistant to either quinolone family or second-line anti-TB injectable drugs. XDR-TB was defined as MDR-TB resistant to any member of the quinolone family and at least one of the remaining second-line anti-TB injectable drugs [3].

#### Treatment outcomes

All patients were following the standard treatment outcomes. Standard WHO outcome definitions were used to detect MDR TB including cure, treatment completion, treatment failure, causes of death, default, and transferring out [16, 17]. Successful outcomes and poor outcomes were defined as cure or treatment completion failure or death, respectively. These were considered as known outcomes, whereas unknown outcomes included default, transferring, or continuing treatment.

#### DNA extraction and sequencing

The crude DNA was extracted from freshly harvested bacteria [18]. The cultured bacteria was extracted from the surface of L-J medium were suspended in 500 μL Tris-EDTA (TE) buffer and heated in a 95 °C water bath for 30 min. The genomic DNA was used as template for amplification. The *pnc*A gene was amplified with the following primers: *pnc*A-F 5′-GTCGGTCATGTTCGCGATCG-3′ and *pnc*A-R 5′- GCTTTGCGGGCGAGCGCTCCA-3′ [19]. The 50 μL PCR mixture was prepared as follows: 25 μL 2 × GoldStar MasterMix (CWBio, Beijing, China), 5 μL of DNA template, and 0.2 μM of each primer set. The amplifications of *pnc*A was performed using the following conditions: 5 min of denaturation at 94 °C followed by 35 cycles (in which each cycle consisted of 94 °C for 1 min, 58 °C for 1 min, and 1 min of extension at 72 °C) and a final extension of 72 °C for 5 min. PCR products were carried out at Personalbio company (Shanghai, China). Gene polymorphisms were aligned with *pnc*A of reference strain H_37_RV (ATCC) using DNAstar MegAlign (version 7.1) software.

#### Genotyping

Members of the strains of Beijing family were identified by the RD105 multiplex PCR [20].

### Statistical analysis

The percentages between PZA-resistant and PZA-susceptible MDR strains resistant to SM and others were compared and analyzed by Crosstabs and Chi square test. The sensitivity and specificity of different methods were examined by Wilson score confidence interval method. Results were considered statistically significant at a two-tailed level of 0.05. Statistical analysis were conducted using SPSS 21.0 (SPSS., USA).

## Results

### Demographic characteristics and drug susceptibility profiles

Totally, 110 (8.3%) out of 1325 clinical isolates were identified as MDR-TB, including 29.1% pre-XDR and 6.4% XDR. Around 70.0% strains were isolated from male patients (Table 1). The average age of the 110 MDR-TB patients was 46.4 years (range 19–85 years). Around 40.9% isolates were from new cases. Among 110 MDR-TB clinical isolates, 59.1, 47.3, 30.0, 30.0, 8.2, 7.3, 3.6, 1.8 and 5.5% were resistant to SM, EMB, OFLX, LVX, KAN, AMK, CAP, PTO and PAS, respectively.

**Table 1:** Risk factor associate with pyrazinamide resistance among 110 MDR isolates

Characteristics	No.(%) of isolates	No.(%) of isolates		*χ*^2^	*P* _value
		PZA^R^	PZA^S^		
	(*n* = 110)	(*n* = 65)	(*n* = 45)		
Sex					
Male	77 (70.0)	41 (63.1)	36 (80.0)	–	0.057
Female	33 (30.0)	24 (36.9)	9 (20.0)	3.626	–
Age group					
<30	19 (17.3)	9 (13.9)	10 (22.2)	–	–
30–59	61 (55.4)	37 (56.9)	24 (53.3)	1.047	0.306
≥ 60	30 (27.3)	19 (29.2)	11 (24.5)	1.211	0.271
Permanent resident					
Yes	58 (52.7)	38 (58.5)	20 (44.5)	–	–
No	52 (47.3)	27 (41.5)	25 (55.5)	2.096	0.148
Treatment history					
New case	45 (40.9)	21 (32.3)	24 (53.3)	–	–
Re-treated	65 (59.1)	44 (67.7)	21 (46.7)	4.863	0.027
Treatment outcome					
Successful	42 (38.2)	19 (29.2)	23 (51.1)	–	–
Poor	68 (61.8)	46 (70.8)	22 (48.9)	5.393	0.020
Cavity					
Yes	65 (59.1)	39 (60.0)	26 (57.8)	–	–
No	45 (40.9)	26 (40.0)	19 (42.2)	0.054	0.816
Initial sputum smear					
Negative	7 (6.4)	6 (9.2)	1 (2.2)	–	–
Positive	103 (93.6)	59 (90.8)	44 (97.8)	2.192	0.279
Genotype					
Non-Beijing family	23 (20.9)	14 (21.5)	9 (20.0)	–	–
Beijing family	87 (79.1)	51 (78.5)	36 (80.0)	0.038	0.845
*Pnc*A gene mutation					
Yes	56 (50.9)	54 (83.1)	2 (4.4)	–	
No	54 (49.1)	11 (16.9)	43 (95.6)	65.787	0.000
Resistance to					
SM	65 (59.1)	44 (67.7)	21 (46.7)	4.863	0.027
EMB	52 (47.3)	37 (56.9)	15 (33.3)	5.937	0.015
OFLX	33 (30.0)	28 (43.1)	5 (11.1)	12.939	0.000
LVX	33 (30.0)	28 (43.1)	5 (11.1)	12.939	0.000
KAN	9 (8.2)	6 (9.2)	3 (6.7)	0.233	0.898
AMK	8 (7.3)	6 (9.2)	2 (4.4)	0.903	0.564
CAP	4 (3.6)	2 (3.1)	2 (4.4)	0.142	1.000
PTO	2 (1.8)	1 (1.5)	1 (2.2)	0.070	1.000
PAS	6 (5.5)	1 (1.5)	5 (11.1)	4.725	0.081
Pre-XDR	32 (29.1)	25 (38.5)	7 (15.6)	6.764	0.009
XDR	7 (6.4)	6 (9.2)	1 (2.2)	1.174	0.279

Abbreviations: R, resistant; S susceptible; SM, streptomycin; EMB, ethambutol; OFX, ofloxacin; LFX, levofloxacin; KAN, kanamycin; AMK, amikacin; CAP, capreomycin; PTO, protionamide; PAS, para-amino salicylic acid; Pre-XDR, pre-extensively extensive drug resistance; XDR, extensive drug resistance

With regard to the treatment history and treatment outcomes among MDR-TB cases, the percentage of re-treated MDR-TB patients in the PZA-resistant group was significantly higher than those in the PZA-susceptible group (*P* = 0.027), and the percentage of treatment successful patients in PZA-susceptible group was significantly higher than in the PZA-resistant group(*P* = 0.020).

The resistance phenotypic of other drugs between PZA-resistant and PZA-susceptible groups were further examined (Table 1). The resistance of streptomycin-(*P* = 0.027), ethambutol-(*P* = 0.015), ofloxacin-(*P*<0.001), levofloxacin-(*P*<0.001) and pre-XDR-(*P* = 0.009) were more frequently detected among PZA-resistant groups compared with PZA-susceptible groups.

### Mutations in the *pnc*A gene

Totally, 50.9% MDR-TB isolates were observed a mutation located in the *pnc*A gene, including 91.1% of single nucleotide substitutions and 8.9% of frame-shift mutation.

Great mutant diversity in *pnc*A gene was observed and 40 different mutant types conferred PZA resistance among MDR strains in Ningbo (Table 2). Two PZA-susceptible isolates harbored a genetic mutation in *pnc*A gene, including 1 strains in codon 8 and 1 strains in codon 76. Considering the phenotypic PZA susceptibility as a gold standard, detection of mutation in *pnc*A gene exhibited a sensitivity of 83.1% and a specificity of 95.6% (Table 3).

**Table 2:** Mutations of *PncA* gene among MDR-TB isolates

Nucleotide position	Codon Change	A.A change	Mutation Type	TBDReaMDB	GMTV Database	No. of isolates
T14 > G	5. ATC/AGC	ILE5SER	Non-synonymous	Unreported	Unreported	2
T17 > C	6. ATC/ACC	ILE6THR	Non-synonymous	Unreported	Reported	2
G19 > T	7.GTC/TTC	VAL7PHE	Non-synonymous	Reported	Reported	1
T20 > G	GTC/TGC	VAL7 GLY	Non-synonymous	Reported	Reported	3
A23 > G	8. GAC/GGC	ASP8GLY	Non-synonymous	Reported	Reported	1
A29 > C	10. CAG/CCG	GLN10PRO	Non-synonymous	Reported	Reported	2
A35 > C	12. GAC/GCC	ASP12ALA	Non-synonymous	Reported	Reported	1
T40 > G	14. TGC/GGC	CYS14GLY	Non-synonymous	Unreported	Unreported	1
T56 > G	19.CTG/CGG	LEU19ARG	Non-synonymous	Reported	Unreported	1
G71 > A	24. GGC/GAC	GLY24ASP	Non-synonymous	Unreported	Reported	1
T100 > G	34. TAC/GAC	TYR34/ASP	Non-synonymous	Unreported	Unreported	1
A139 > G	47. ACC/GCC	THR47ALA	Non-synonymous	Reported	Reported	1
A142 > G	48. AAG/GAC	LYS48GLU	Non-synonymous	Reported	Reported	1
C151 > T	51. CAC/TAC	HIS51TYR	Non-synonymous	Reported	Reported	2
A170 > C	57. CAC/CCC	HIS57PRO	Non-synonymous	Reported	Unreported	1
C184 > A	62. CCG/ACG	PRO62THR	Non-synonymous	reported	Unreported	1
C206 > G	69. CCA/CGA	PRO69ARG	Non-synonymous	Unreported	Unreported	1
A226 > C	76. ACT/CCT	THR76PRO	Non-synonymous	Reported	Reported	1
A287 > C	96. AAG/ACG	LYS96/THR	Non-synonymous	Reported	Reported	1
294–95	Deletion T	FRAMSHIPT	FRAMSHIPT	Unreported	Unreported	1
C312 > A	104. AGC/AGA	SER104ARG	Non-synonymous	Reported	Reported	1
G314 > A	105. GGC/GAC	GLY105ASP	Non-synonymous	Reported	Reported	1
A329 > G	110. GAC/GGC	ASP110GLY	Non-synonymous	reported	Unreported	1
A345 > C	115. CCA/CCC	PRO115PRO	Synonymous	Unreported	Unreported	1
T347 > G	116. CTG/CGG	LEU116ARG	Non-synonymous	Reported	Reported	1
C372 > A	124. GGC/GGA	GLY124GLY	Synonymous	Unreported	Unreported	1
T374 > G	125. GTC/GGC	VAL125GLY	Non-synonymous	Reported	Reported	1
392–93	Insertion GG	FRAMSHIPT	FRAMSHIPT	Unreported	Unreported	3
G394 > T	132. GGT/TGT	GLY132CYS	Non-synonymous	reported	Unreported	1
C401 > T	134. GCC/GTC	ALA134VAL	Non-synonymous	Reported	Reported	1
A403 > C	135. ACC/CCC	THR135PRO	Non-synonymous	Reported	Reported	1
A407 > C	136. GAT/GCT	ASP136ALA	Non-synonymous	Unreported	Reported	2
A410 > C	137. CAT/CCT	HIS137PRO	Non-synonymous	Unreported	Unreported	3
G415 > C	139. GTG/CTG	VAL139LEU	Non-synonymous	reported	Unreported	1
T416 > G	139. GTG/GGG	VAL139ALA	Non-synonymous	Reported	Reported	2
A424 > G	142. ACG/GCG	THR142ALA	Non-synonymous	reported	Unreported	3
T464 > G	155. GTG/GGG	VAL155GLY	Non-synonymous	Reported	Reported	1
T470 > G	157. GTG/GGG	VAL157GLY	Non-synonymous	Unreported	Unreported	1
A478 > C	160. ACA/CCA	THR160PRO	Non-synonymous	Unreported	Reported	2
497–98	Insertion G	FRAMSHIPT	FRAMSHIPT	Unreported	Unreported	1
G538 > T	180. GTC/TTC	VAL180PHE	Non-synonymous	Reported	Unreported	2

Abbreviations: MDR, multi drug resistance; TBDReaMDB, Tuberculosis Drug Resistance Mutation Database; GMTV, Genome-wide *Mycobacterium tuberculosis* variation

**Table 3:** Performance of *Pnc*A mutations for predicting PZA susceptibility

Mutation in *Pnc*A	PZA susceptibility^a^		Total	Sensitivity^b^ (95% CI, %)	Specificity^b^ (95%CI, %)	PPV (95%CI, %)	NPV (95%CI, %)
	R	S					
Yes	54	2	56	83.1	95.6	96.43	79.6
No	11	43	54	(71.3–90.9)	(83.6–99.2)	(86.6–99.4)	(66.1–88.9)
Total	65	45	110				

^a^*R*, resistant, *S* susceptible, *PPV* positive predictive value, *NPV* negative predictive value, *CI* confidence interval

^b^The sensitivity and specificity were examined by Wilson score confidence interval method

## Discussion

PZA as an important first-line anti-tuberculosis drug plays a crucial role in the therapeutic treatment of MDR-TB [21, 22]. Considering the unique effect of PZA, the detection of PZA among MDR-TB is a significant factor for initiation of PZA in the therapy regimens for these refractory patients [23, 24]. This study showed that the PZA resistance rate among MDR-TB in Ningbo was 59.1%, which higher than those in Zhejiang Province (43.1%) [25], Shanghai (38.5%) [26], Thailand (49.0%) [27], United states (38.0%) [28], and similar to a recent result from Beijing (57.7%) [29].

About 60% MDR patients received previous anti-TB therapy with PZA in our study, which is significantly higher than the national level (21.8%) [4]. Our results demonstrated that the high frequency of PZA resistance may contribute to the high rate of re-treated TB patients. Therefore, we proposed to diminish the role of PZA in the treatment for MDR-TB in Ningbo. It was necessary to formulate a suitable regimen by detecting PZA resistance before using of PZA for treatment of MDR-TB cases.

Bacterial species induces the production of oxygen radicals which result in high frequency mutagenesis by exposing to antimicrobial agents, including RIF, FQ and the aminoglycosides [30, 31]. A recent report from Alame-Emane and colleagues reveled that PZA resistance in *M.tuberculosis* arised after RIF and fluoroquinolone (FQ) resistance [32]. In line with our findings, one recent result from our observation was that there were high correlation between PZA resistance and several other drugs resistance, including SM, EMB, OFLX and LVX. Regarding long duration of anti-TB treatment, MDR bacteria existed more genetic mutations which may be responsible for the potential cross resistance between PZA and other drugs in our study. It is necessary to perform PZA susceptibility testing for proper management of MDR-TB with regimen containing PZA. However, traditional PZA drug susceptibility testing is not routinely performed due to the requirement of harshly acidic environment which many isolates of *M.tuberculosis* failed to grow [33]. As an alternative testing to predict the PZA susceptibility in *M.tuberculosis,* Molecular method based on detecting the mutation in *pnc*A is essential [34, 35].

Previous study showed that genetic mutations constituted the most import mechanism conferring drug resistance in *M.tuberculosis* [36]. The result from our study suggested that genetic alternations in *pnc*A confer 83.1% of PZA resistance among MDR-TB in Ningbo. Previous studies demonstrated a diverse prevalence of *pnc*A mutation among PZA resistance isolates in different regions, ranging from 45.7% in Brazil [37], 70.6% in Iran [38], 75.0% in Thailand [27], 78.0% in Zhejiang [25], 84.6% in Southern China [39], and 94.1% in Sweden [40]. Additionally, *pnc*A mutations exhibited great diversity in our study, although *pnc*A mutations were not statistically significant between 2 genotypes - Beijing family and non-Beijing family. Hence, DNA sequencing of the entire *pnc*A was more effective for detection of PZA resistance rather than the routine methods by covering the mutant hot-sports. Moreover, other reports suggested that many MDR-TB and XDR-TB outbreaks were caused by strains of the Beijing family which had an increased tendency to develop drug resistance [41, 42]. However, the data from our study showed that there was no significant difference between PZA-resistant and PZA-susceptible group in genotype. The genetic background capable of accumulating resistance was not observed in our study. The effect of PZA resistance during chemotherapy on treatment outcomes are still lack of evidence [43, 44]. Our subjects were compared with successful and poor treatment outcomes in terms of resistance to PZA in this study. According to our observations, treatment outcomes were significantly better with PZA susceptible MDR patients, suggest the need for PZA resistance test to optimize treatment.

To our knowledge, this study was the first investigation on phenotypic and molecular characterization of PZA resistance among multidrug-resistant *Mycobacterium tuberculosis* (MDR-TB) isolates in Ningbo. Nonetheless, some limitations of this study need to be considered. First, the small sample size cannot be representative for TB patients in the whole Ningbo. Second, only MDR-TB isolates were detected in our study. Therefore, further experiments need to be analyzed the contributions of *pnc*A mutations to PZA resistance in non-MDR isolates. However, this study provides critical evidence to diagnose PZA resistance and help guide the treatment with PZA for MDR-TB patients in this region with high MDR-TB burden.

## Conclusion

The sequencing of the *pnc*A gene in our study provided rapid and reliable information against PZA susceptibility for MDR-TB isolates in Ningbo. The PZA-resistant isolates in MDR-TB were likely to have concomitant resistance to streptomycin, ethambutol, ofloxacin, levofloxacin and pre-XDR. DNA sequencing of the entire *pnc*A was more effective to predict PZA resistance rather than the routine methods by covering the mutant hotspots because of its high degree of diversity in *pnc*A gene. The high prevalence of PZA resistance among MDR suggested that we should adjust PZA in a timely and accurate manner in a treatment regimen for MDR-TB in this setting with TB burden. Future study should aim to clarify the potential contributions of other gene mutations to PZA resistance caused by various medication treatments.

## Data Availability

Please contact the corresponding author for data requests.

## Abbreviations

TB: Tuberculosis
WHO: World Health Organization
MDR: Multidrug-Resistant
SM: Streptomycin
EMB: Ethambutol
OFX: Ofloxacin
LFX: Levofloxacin
KAN: Kanamycin
AMK: Amikacin
CAP: Capreomycin
PTO: Protionamide
PAS: Paza-aminosalioylate
Pre-XDR: Pre-extensively extensive drug resistance
XDR: Extensive drug resistance
